# Anti-Müllerian hormone: biology and role in endocrinology and cancers

**DOI:** 10.3389/fendo.2024.1468364

**Published:** 2024-09-16

**Authors:** Marek Gowkielewicz, Aleksandra Lipka, Wojciech Zdanowski, Tomasz Waśniewski, Marta Majewska, Carsten Carlberg

**Affiliations:** ^1^ Department of Gynecology and Obstetrics, School of Medicine, Collegium Medicum, University of Warmia and Mazury in Olsztyn, Olsztyn, Poland; ^2^ Institute of Oral Biology, Faculty of Dentistry, University of Oslo, Oslo, Norway; ^3^ Department of Human Physiology and Pathophysiology, School of Medicine, University of Warmia and Mazury in Olsztyn, Olsztyn, Poland; ^4^ Institute of Animal Reproduction and Food Research, Polish Academy of Sciences, Olsztyn, Poland; ^5^ School of Medicine, Institute of Biomedicine, University of Eastern Finland, Kuopio, Finland

**Keywords:** AMH, AMHR2, neuroendocrinology, oncology, signal transduction

## Abstract

Anti-Müllerian hormone (AMH) is a peptide belonging to the transforming growth factor beta superfamily and acts exclusively through its receptor type 2 (AMHR2). From the 8^th^ week of pregnancy, AMH is produced by Sertoli cells, and from the 23^rd^ week of gestation, it is produced by granulosa cells of the ovary. AMH plays a critical role in regulating gonadotropin secretion, ovarian tissue responsiveness to pituitary hormones, and the pathogenesis of polycystic ovarian syndrome. It inhibits the transition from primordial to primary follicles and is considered the best marker of ovarian reserve. Therefore, measuring AMH concentration of the hormone is valuable in managing assisted reproductive technologies. AMH was initially discovered through its role in the degeneration of Müllerian ducts in male fetuses. However, due to its ability to inhibit the cell cycle and induce apoptosis, it has also garnered interest in oncology. For example, antibodies targeting AMHR2 are being investigated for their potential in diagnosing and treating various cancers. Additionally, AMH is present in motor neurons and functions as a protective and growth factor. Consequently, it is involved in learning and memory processes and may support the treatment of Alzheimer’s disease. This review aims to provide a comprehensive overview of the biology of AMH and its role in both endocrinology and oncology.

## 1 Introduction

**Figure 1:**
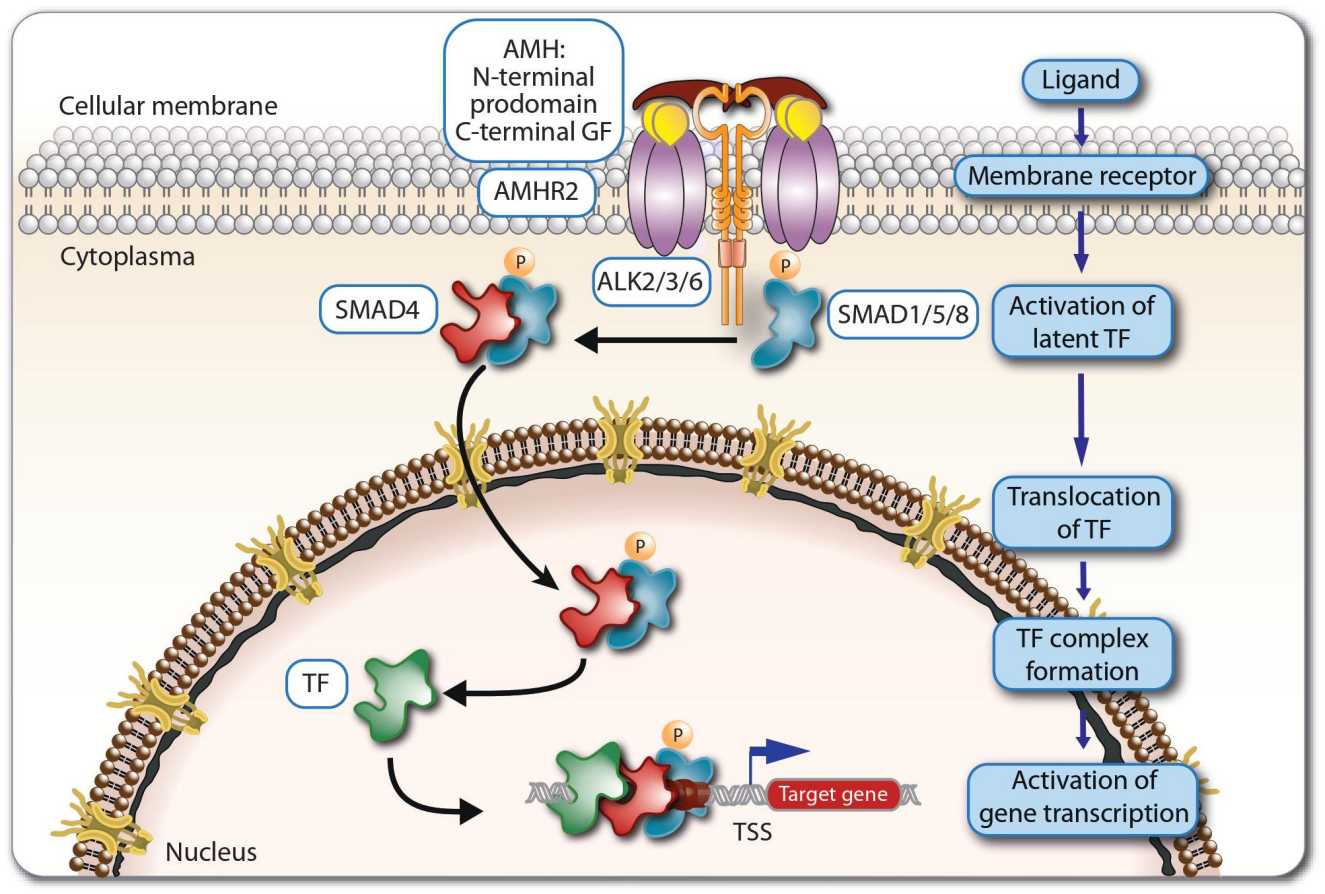
Principles of AMH signaling. Dimeric AMH peptides activate the receptor AMHR2, which then together with ALKs activate the regulatory SMAD proteins 1, 5 and 8. The latter translocate to the nucleus and act together with SMAD4 and other supporting transcription factors (TFs) as regulators of the expression of AMH target genes.

AMH, also known as a Müllerian inhibiting factor or Müllerian inhibiting substance, has a mani-fold and complex effect on the development and the function of a variety of human tissues. AMH is a glycoprotein belonging to the transforming growth factor beta (TGFβ) superfamily (1). This family of signaling proteins includes 32 other peptides, such as activins, inhibin A and B, bone morphogenic factors (BMPs) and growth differentiation factors (GDFs) like myostatin (2, 3). The AMH protein composed of a N-terminal prodomain and a C-terminal growth factor (GF) domain (**Figure 1**), which is encoded by a gene consisting of 5 exons and 4 introns, located on the short arm of human chromosome 19 (4). The regulatory regions of the *AMH* gene contain binding sites for multitude of transcription factors, such as SOX9 (SRY-box transcription factor 9) and NFκB (nuclear factor kappa B) (5, 6).

There are seven type 1 and five type 2 receptors for all 33 members of TGFβ superfamily (5, 7). AMH is binds exclusively to the type 2 receptor - AMHR2 (8). Therefore, only cells expressing AMHR2 are able to respond to direct AMH stimulation (9). The AMHR2 protein is encoded by a gene located on the long arm of human chromosome 12, comprising 11 exons and 10 introns. The first three exons encode the extracellular domain, the fourth exon the transmembrane domain, while the remaining seven exons encode the intracellular serine/threonine kinase domain (10). The *AMHR2* gene responds to similar regulatory signals as the *AMH* gene (5). Due to the orientation of the N-terminus to the extracellular space, AMHR2 is classified as a type 1 membrane protein (11). After ligand binding, AMHR2 acts as a transmembrane serine-threonine kinase and activates AMH type 1 receptors (5, 11, 12) (**Figure 1**). The latter are signal enhancement molecules, which also exhibit serine-threonine kinase activity. The AMH signaling pathway uses three types of activin receptor-like kinases (ALKs), ALK2, ALK3, ALK6, in different types of tissues, with ALK2 and ALK3 acting as positive regulators in signal transduction and ALK6 mainly as an inhibitor (5, 12–14). The AMH-AMHR2-ALK complex phosphorylates regulatory members of the SMAD (SMA- and MAD-related protein) family, SMAD 1, 5 and 8, which after translocating to the nucleus, they regulate together with SMAD4 and other supporting transcription factors the expression of AMH target genes (3, 5, 12, 15, 16).

Multiple studies indicated that AMH is expressed in Sertoli cells of the testes, granulosa cells (GCs) of the ovaries (preantral and small antral follicles), the endometrium of women in the reproductive age, motoneurons, gonadotropin-releasing hormone (GnRH) neurons and the hippocampus as well as in endometrial cancer (EC), sex cord-stromal tumors and granulosa cell tumors (17–24). In addition, traces of AMH are found in skeletal muscles, the sciatic nerve, the spinal cord and the mouse brain (23). *In vitro* studies and animal models revealed that AMH induces cell cycle inhibition and apoptosis in some cancer cell lines (25–27). Moreover, AMH also shows an additive or synergistic effect in combination with typical chemotherapeutic agents in serous ovarian cancer cells that express AMHR2 (28). A large number of reports found AMHR2 expression in cells of the Müllerian ducts, ovarian follicles (preantral and small antral), the pituitary gland, the hypothalamus, the endometrium, the adrenal glands, lactiferous ducts, Leydig cells, the prostate, motor neurons, the hippocampus and some of the human cancers that include endometrial, ovarian, prostate, breast, and cervix (21, 23, 25–27, 29–36). Based on the epithelial-mesenchymal transition (EMT) process, AMHR2 could be expressed also in some solid tumors (29, 37–39). So far, *AMHR2* expression has been confirmed, among others, in non-small cell lung cancer and ocular melanoma (29, 40).

**Figure 2:**
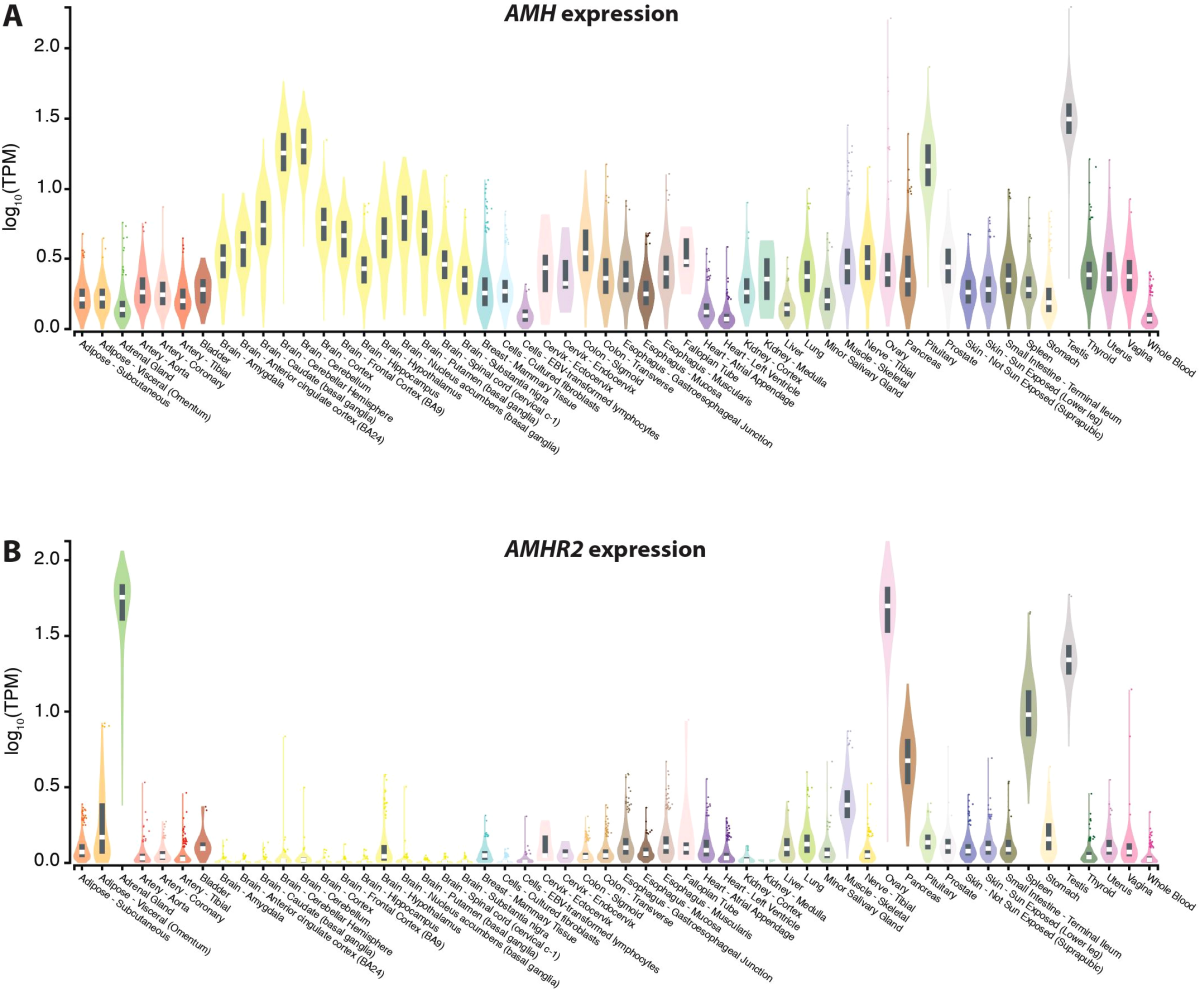
Gene expression based on data of the GTEx project. Expression of the genes *AMH* **(A)** and *AMHR2* **(B)** in 54 different human tissues. Normalized RNA sequencing data are shown in TPM (transcripts per million), where all isoforms were collapsed into a single gene. Box plots display the median as well as 25^th^ and 75^th^ percentiles. Points indicate outliers that are 1.5 times above or below interquartile range. Data are based on GTEx analysis release V8 (dbGaP Accession phs000424.v8.p2).

The publicly available dataset (https://gtexportal.org) of the GTEx (Genotype-Tissue Expression) project is the gold standard for comparing tissue-specific gene expression (41). Based on 54 tissues obtained from 948 post-mortem donors the expression of the *AMH* gene is highest in testis, pituitary gland and cerebellum, but most other investigated tissues also show some expression of the gene (**Figure 2A**). In contrast, the expression of the *AMHR2* gene is far more restricted to adrenal gland, ovary, testes, spleen and pancreas (**Figure 2B**).

**Figure 3:**
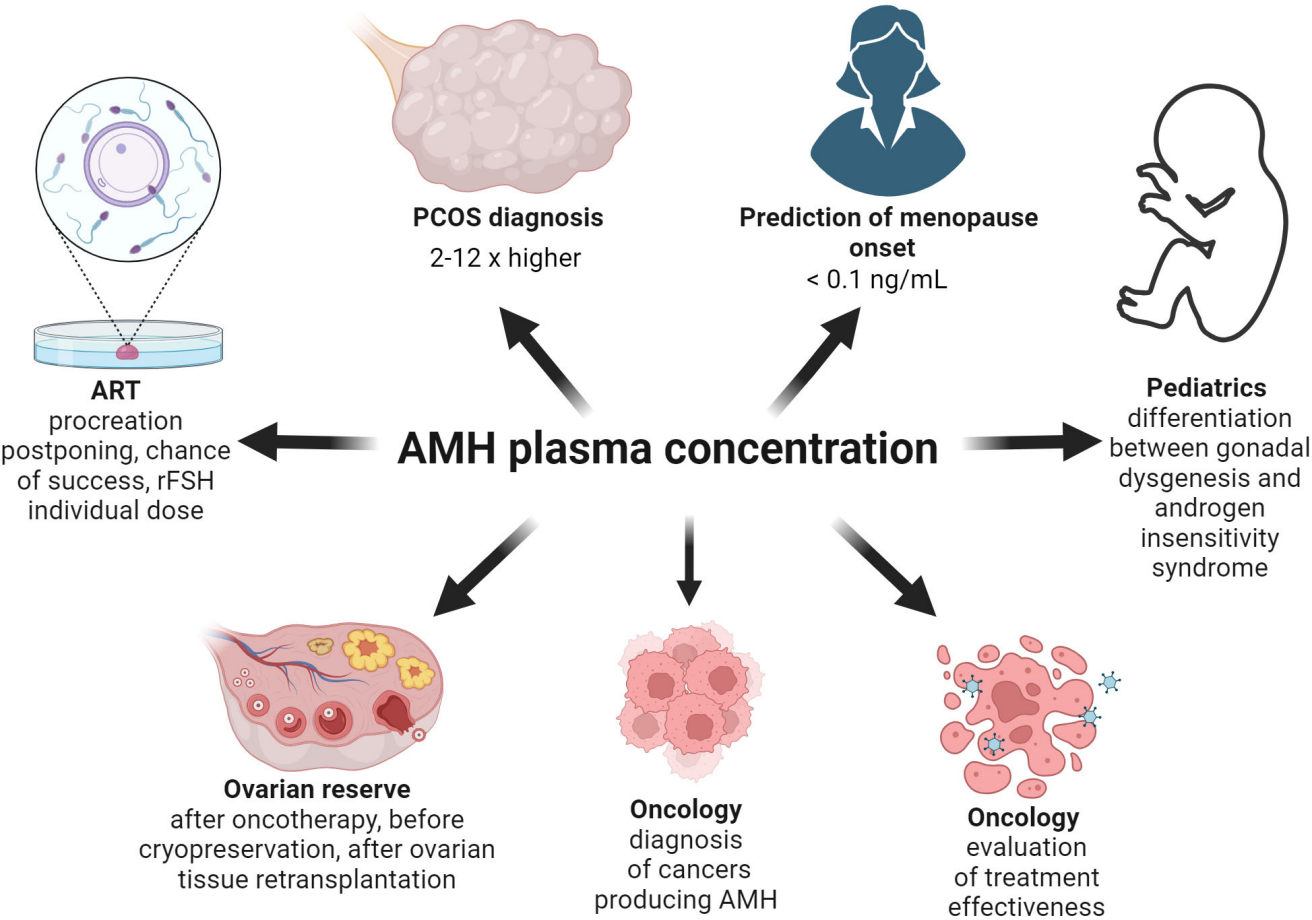
The utility of the assessment of AMH concentration on the different fields of medicine. As the AMH concentration increases 2-12 times in the PCOS women group, its level should be taken into account while diagnosing PCOS. Based on mathematical modeling of the decreasing AMH levels with age, a concentration below 0.1 ng/mL means menopause. The AMH level enables a correct diagnosis between gonadal dysgenesis and androgen insensitivity syndrome in 46, XY individuals with incorrect appearance of their genitalia. AMH concentration facilitates diagnostics and helps evaluate the treatment of the cancers producing AMH. As AMH level is the best marker of ovarian reserve, it should be assessed before and after oncotherapy, before cryopreservation and after retransplantation of ovarian tissue. Moreover, in the infertility treatment clinic the AMH level is useful as the factor evaluating the chance of live birth and allowing the use of the appropriate dose of recombinant FSH.

The serum concentration of AMH in healthy women of the reproductive age is in the range of 1.5-4.0 ng/mL (42). Tumors originating from sex cord cells and ovarian GCs produce AMH and in those cases the serum concentration often exceeds the reference value up to 1000 times (17, 43) (**Figure 3**). For example, a AMH concentration of 3205 ng/mL was found in a patient diagnosed with metastatic sex cord tumor (17). It is worth emphasizing here that there are several commercial laboratory tests (kits) for assessing AMH concentration. They differ significantly in sensitivity, intra-assay and inter-assay variation coefficient (44). Hence, when interpreting AMH test results in different studies or meta-analyses, it is worth considering which ELISA (enzyme-linked immunosorbent assay) kit was used (44). This is also clinically important, for example when comparing AMH rate of change. It would be best to do this with the same laboratory test. Decreasing AMH levels are the evidence of the effectiveness of treatment in this type of cases (**Figure 3**). Interestingly, increased levels of AMH do not cause any toxic effects (45). For this reason, it is postulated that AMH has a remarkably beneficial profile and may be useful in the treatment of tumors expressing AMHR2 (45). The aim of this narrative review is to systematize knowledge about the expression of AMH and AMHR2 in various tissues, in order to gather information on the mechanism of action of AMH in physiological and various medical conditions, especially concerning malignant tumors.

## 2 Methods

A literature review on the discussed topic was conducted by searching for keywords/phrases in publicly available databases as: PubMed, Google Scholar, ScienceDirect. Those keywords included: “anti-Müllerian hormone receptor type 2,” “AMHRII” or “AMHR2,” “anti-Müllerian hormone receptor type 1, “anti-Müllerian hormone,” “AMH,” “MIS”, “anti-Müllerian hormone and cancer,” “anti-Müllerian hormone and PCOS,” “anti-Müllerian hormone and endometriosis,” “anti-Müllerian hormone and ovarian function,” “anti-Müllerian hormone and pituitary gland function” “anti-Müllerian hormone and hypothalamus”, “anti-Müllerian hormone and artificial reproductive technology”, “anti-Müllerian hormone and menopause”. Only articles in English and Polish were taken into account. Additionally, publications were searched for manually on the basis of references provided in the selected papers. The data analyzed came from published articles and a chapter of one book regarding the main theme and related topics.

## 3 AMH in fetal life, childhood and puberty

**Figure 4:**
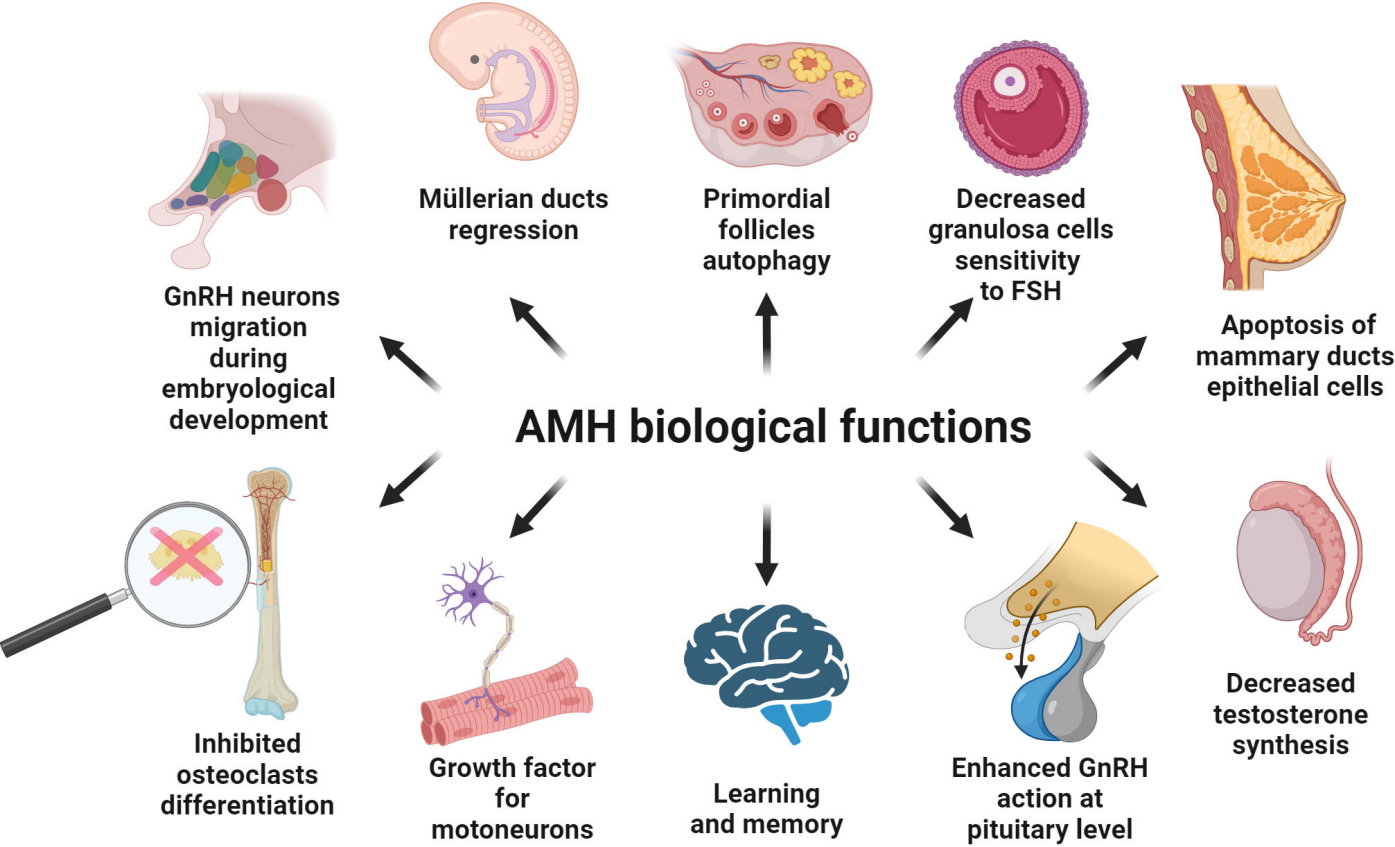
Known biological function of AMH in the different types of the tissues. Details are described in the text; below are the main facts. AMH is involved in the migration of GnRH neurons to the target site at the early stages of human embryological development. AMH is produced by Sertoli cells and inhibits the development of Müllerian ducts. AMH synthetized by granulosa cells of the ovary negatively influences FSH action at the level of the ovary. AMH is engaged in the apoptosis of the epithelial cells of mammary ducts after the lactation period. AMH inhibits testosterone synthesis and positively impacts the secretion of gonadotropins under the influence of GnRH. AMH is engaged in memory and learning processes in the hippocampus. AMH is present in motor neurons and acts as a protective and growth factor. AMH inhibits RANKL-dependent osteoclast differentiation.

Mammals bi-directionally differentiate their reproductive organs during early embryonic development. The Müllerian ducts give rise to the upper third of the vagina, the cervix, the body of the uterus, uterine tubes and the ovarian tunica albuginea (25). The Wolffian ducts differentiate into seminal vesicles, the *vas deferens* and the epididymis (46). The genesis of one set of ducts, along with the atrophy of the other, is controlled by hormones AMH and testosterone. In female embryos, the lack of testosterone is responsible for the atrophy of the Wolffian ducts. In male embryos, the presence of AMH results in the regression of structures derived from the Müllerian ducts (46). This process acts mainly *via* intracytoplasmic accumulation of β-catenin in mesenchymal cells, which finally activates apoptosis of type I and type II in epithelial cells (12, 47, 48), and takes place in the cranial-caudal direction so that the density of AMHR2 increases (47, 48). Degeneration of the Müllerian ducts ends with EMT of epithelial cells, which previously have not entered the apoptosis process (47, 48). The regression of Müllerian ducts occurs in human male embryos after the 8^th^ week of gestation (47, 49–52) (**Figure 4**). In fetal life, AMH also affects other types of cells. It can have disadvantageous effects on the maturation of type II pneumocytes, which explains the more frequent occurrence of respiratory distress syndrome in prematurely born male infants (53).

AMH is synthesized both in the testes and the ovaries but with a different timing. During development of the male fetuses, AMH is expressed in the Sertoli cells of gonads as early as 8 weeks of pregnancy (48 days post conception) (54–56), and then it reaches high serum values (~50 ng/mL) (45, 57). In postnatal males, the AMH level is inversely proportional to the testosterone level (58). Thus, AMH secretion is under negative control of testosterone, while the expression of androgen receptor increases in Sertoli cells during childhood and puberty (5, 55, 59). During the first month after birth, the AMH serum concentration increases about 1.9-fold and within the next 11 months it still increases, yet more slowly. Starting from the first year of life, the AMH level slowly decreases, reaching half of its maximal value between age 6 and 8, till puberty (60). At that point, the AMH level drops to the baseline values (57). Male mice with elevated AMH levels simultaneously present Leydig cell hypoplasia, decreased testosterone level and, as a consequence, incorrect virilization (61). Conversely, mice with a null mutation of the *AMH* or *AMHR2* gene exhibit Leydig cell hyperplasia (62). Since AMHR2 is expressed on the surface of Leydig cells, AMH inhibits the production of testosterone (34, 63) (**Figure 4**).

In newborns or young boys possessing correct male karyotype but presenting symptoms of incorrect virilization, the AMH level allows the distinction of gonadal dysgenesis and androgen insensitivity syndrome (64–66) (**Figure 3**). In boys with the persistent Müllerian ducts, AMH function is compromised due to defects or mutations within the *AMH* or *AMHR2* gene, so its serum level is helpful in making a correct diagnosis (64, 67, 68) (**Figure 3**). Also, in individuals with a 46,XX karyotype but pathological appearance of internal and external genitalia, AMH concentration provides information concerning the presence of testicular tissue in those cases (64, 69).

In female fetuses, *AMH* gene expression starts in GCs of preantral ovarian follicles (35) at 36 weeks of gestation (56), and AMH proteins are not detected in the serum until the 37^th^ week of gestation (60). In contrast, a more recent study indicated that *AMH* gene expression on GCs started from 23 weeks of gestation and serum AMH levels are detectable in female newborns born after 25 weeks and 6 days of pregnancy (70). Precise regulation of the AMH synthesis and its concentration is necessary to maintain proper oocyte resources. Female mice with AMH-chronic expression during pregnancy (at a similar level to male fetuses) had fewer germ cells at birth than those with normal AMH levels, entirely losing them 16 days after parturition (61). Primordial follicles of AMH^-/-^ mice were recruited earlier for maturation and had more preantral and antral follicles than wild type mice, but their pool was depleted significantly earlier (71).

Shortly after parturition, the AMH level drops and increases again around the age of 2 years, to decline once again between the age of 8 and 12 (72). At puberty, the AMH level increases, reaching the baseline values and peaks at 24.5 years of age. From this point, the AMH concentration gradually lowers to a non-detectable level at menopause (42, 45, 72). Importantly, the concentration of AMH does not depend on the phase of the menstrual cycle (73, 74). AMH level in the short period of time, which is the menstrual cycle, seems to be more or less constant. AMH is produced by several cohorts of developing follicles at different stages of maturation in both ovaries, which are the subject to specific cyclic regulations. However, some researchers have noticed a dependence of AMH concentration on the phase of the cycle (75). A meta-analysis conducted on 733 healthy women showed that the difference in AMH concentration between the follicular and luteal phase is about 11.5% in favor of the follicular phase (44). This seems to be important in the context of preconception counselling in the infertility clinics or when planning *in vitro* fertilization (IVF) procedures (44). Moreover, only GCs of preantral and small antral ovarian follicles produce AMH and exhibit AMHR2 (35). AMH reduces the activity of the aromatase enzyme, and its production is inhibited by the increasing concentration of estrogen (71, 76).

Ovarian reserve (OR) is a pool of primordial follicles (77–79), and AMH is one of the factors to estimate the remaining OR (**Figure 3**). Although AMHR2 seems not be expressed in primordial follicles, AMH may have an autocrine effect on GCs of preantral and small antral ovarian follicles. There was a simplified assumption that those follicles under the influence of AMH release factors regulating primordial follicle recruitment (71). It has also been shown that adding AMH to ovarian tissue cells inhibits the growth of primordial follicles (80). It turned out that the regulation of autophagy is responsible for the recruitment of primordial follicles. At various stages of the transformation of primordial follicles towards the ovulatory follicle and then the *corpus luteum*, autophagy plays a significant role (76). Autophagy is a process thanks to which the cell has a chance to repair damaged organelles, maintain homeostasis in unfavorable environmental conditions, remove toxic compounds and possibly directs the cell towards apoptosis (76, 81–84). Furthermore, autophagy in the oocyte reduces oxidative stress and regulates the functionality of the proteome (76, 85, 86). Hence, abnormal autophagy can cause infertility (76, 87).

AMH being secreted by preantral and small antral follicles (up to 8 mm in diameter) as a paracrine factor inhibits the transition of primordial follicles into the primary follicle (71, 76, 88, 89). The effect of AMH is opposite to that of other members of the TGFβ family, such as GDF and BMPs, which promote primordial follicles transition into primary follicle (90). Phosphorylation of the transcription factor FOXO3 (forkhead box O3) promotes the conversion of primordial follicles into primary follicle, while AMH inhibits FOXO3 phosphorylation and induces autophagy in the oocyte (91) (**Figure 4**). However, AMH is not the only signal controlling oocyte autophagy, which is also promoted by inhibition of the PI3K/AKT/mTOR (phosphatidylinositol 3-kinase/AKT serine/threonine kinase/mammalian target of rapamycin) resulting in increased expression of the genes *MAP1LC3B* (microtubule associated protein 1 light chain 3 beta), *BECN1* (beclin 1) and *ATG* (autophagy-related) (76, 92, 93). Interestingly, chronic expression of AMH in early stages of female fetal development may exercise a harmful effect on primordial follicles leading to apoptosis *via* autophagy (76).

## 4 AMH action in the pituitary gland

There seems to be a feedback loop between the gonads and the pituitary gland, in which AMH increases follicle-stimulating hormone (FSH) and luteinizing hormone (LH) synthesis in the pituitary gland, while FSH reduces the production of AMH in the ovaries (94–96) and increases in the testes (5, 6, 55, 64). In males FSH positively controls AMH secretion by activating the *AMH* promoter through phosphorylation of different transcription factors, like NFκB, SF1 (steroidogenic factor 1) and SOX9 (5, 6, 55, 64). FSH may inhibit AMH synthesis in ovarian GCs by increased activity of aromatase and higher concentrations of estradiol, thus stimulating ESR2 (estrogen receptor 2) (95), but this is inconsistent with other studies (97, 98). The discrepancies result largely from the adopted research model, human or animal, *in vivo* or *in vivo* (98). Since AMHR2 is prominently expressed in the pituitary gland, AMH seems to have a regulatory role in the gland (80, 94, 99). For example, FSH serum levels in female *AMH* ^-/-^ mice are significantly lower than in wild type mice (71), while male mice with *AMH* overexpression had elevated plasma levels of LH (8-fold) and FSH (1.5-fold) (34). Together with GnRH, AMH increases the expression of the genes *FSHB* (follicle stimulating hormone subunit beta) and *LHB* (luteinizing hormone subunit beta) in both sexes (94). This is consistent with the suggestion that AMH induces LH secretion by affecting GnRH neurons in the hypothalamus (100) (**Figure 4**). However, AMH has no effect on the expression of the GnRH receptor but GnRH regulates the expression of AMHR2 in the pituitary gland at different moments of the menstrual cycle and before the puberty (94, 99, 101, 102). It is worth adding that in cases of male and female hypogonadotropic hypogonadism which was not caused by a defect in AMH synthesis, increased AMH concentrations were observed compared to the group of healthy people (103, 104). Gonadotropin therapy lowers this elevated AMH concentration (103). On the other hand, in a group of women with idiopathic hypogonadotropic hypogonadism with initially low AMH levels, gonadotropin therapy increases its concentration indicating proper response of ovarian tissue to stimulation (105).

## 5 AMH and the hypothalamus

At early stages of human and murine embryological development AMH is expressed in GnRH neurons (24). By activating the MAPK (mitogen-activated protein kinase) pathway, AMH acts in a paracrine manner and is involved in the migration of GnRH neurons to their target sites (24) (**Figure 4**). Accordingly, mutations of the genes *AMH* or *AMHR2* are associated with hypogonadotropic hypogonadism (24).

AMHR2 is expressed in the cortex hippocampus and hypothalamus of adult mice, as well as in non-neuronal cells belonging to the median eminence, such as endothelial cells and tanycytes (100). Moreover, AMHR2 is expressed in GnRH neurons of murine and human fetuses as well as in adult individuals of those species (24, 100). Specifically, in more than half of GnRH neurons in adult mice and humans AMHR2 expression was found in the preoptic region, including the *organum vasculosum* of the lamina terminalis and in rostral aeras in the septum and diagonal band of Broca. Therefore, approximately 50% of murine GnRH neurons responding to AMH, in both males and females, regardless of the phase of the cycle, although the duration of the response to AMH stimuli is significantly shorter in diestrus animals (100). Interestingly, the secretion of GnRH under the influence of exogenous AMH occurs even after ovariectomy. However, this reaction is specific to AMH, while TGFβ1 has no effect on GnRH release from GnRH neurons (100). Obviously, the secretion of GnRH is not dependent only on AMH (99, 106, 107).

The effect of AMH on GnRH neurons can be very prompt, *e.g.*, when it is administered to the lateral ventricles of female dioestrus mouse, and is not conducted by the canonical SMAD proteins, whose activation takes several hours, but *via* a rapid non-genomic pathway (100). This nontypical route uses AMHR2 signaling and utilizes GnRH receptors in the pituitary gland, since both an ALK 2/3/6 inhibitor and the GnRH antagonist cetrorelix prevents any LH surge (100).

In rats, AMH affects sexual dimorphism of the central nervous system structures (medial preoptic area) and appears to be a regulator of neural network formation, because AMHR2 is expressed in this region (100, 108). Calbindin 1 (CALB1) positive neurons located in the medial preoptic area respond to AMH during fetal life, which correlates with the density of these neurons observed in both sexes in the prepubertal period and in adulthood between *AMH* ^-/-^ and wild type animals (108). These CALB1^+^ neurons play a protective role on dopaminergic neurons *via* the PI3K/AKT/mTOR signaling pathway (109, 110). They also create diversity of social behaviors, fear memory and *Calb1* knockout mutation leads to exhibiting less anxiety in various situations (110, 111). CALB1^+^ neurons change hippocampal excitatory pathway due to early-life stress and disturb stress-related memory (110, 112).

## 6 AMH and other neurons

The proper development of the spinal cord motor neurons and their appropriate response to damaging factors is regulated by a variety of signals. In addition to cardiotrophin-1 (113), TGFβ2 (114) and GDNF (glial cell derived neurotrophic factor) (115) also AMH contributes to it (23). Interestingly, 13-15-day-old mouse fetuses and 5-8-week-old adult mice showed that AMH determines physiological densification of neurons during fetal life. In adult mice, AMH ensures proper autocrine and paracrine functions, especially in the case of neuron damage (23, 116). Accordingly, in motor neuron disorders AMH has a protective effect and could be therapeutically advantageous (23). *AMH* gene expression is highest in in motor neurons with levels comparable to that in Sertoli cells and ovarian GCs (23) (**Figure 4**).

The expression strength of AMHR2 is 30 times higher in motor neurons than in other parts of the brain, the spinal cord or muscles (23). AMHR2 is also a prominently expressed receptor in motor neurons of the spinal cord with levels 40 times higher than the type 2 receptor for TGFβ (23) and 5 times higher than the GDNF receptor (117). Interestingly, there are no gender differences in AMHR2 expression. Thus, AMH can be considered as a neuronal growth factor, since in *in vitro* culture of motor neurons it increased the survival rate of nerve cells and the growth of neurites with a similar effect as the use of classical neuronal growth factor like GDNF (23) (**Figure 4**).

When the level of AMH expression in women with epilepsy that have frequent seizures are compared those that are free from seizures for some period of time and those that are healthy (118), it was found that AMH concentration is higher in the group of women without seizures within the last nine or more months than those with seizures (118). Therefore, AMH may be considered a protective factor against seizures (118).

Interestingly, single nucleotide polymorphisms (SNPs) of the *AMH* gene affect male cognitive function (119). An increased AMH level is connected to worse episodic memory, reaction time and reduced reasoning scores, but has no influence on executive skills and complex processing speed (119). There is a slight negative correlation between the level of AMH and specific characteristics of boys’ autism, such as social interaction and communication. However, total concentrations of AMH in healthy boys compared with boys having autism spectrum disorders are similar although the SMAD pathway may be engaged in autism spectrum disorders (120). On the other hand, AMH levels reflect different drawing skills of boys and girls at the age of 5-6 years, in favor of girls in an inversely dependent manner in comparison to the concentration of AMH (121). This suggests that autistic boys may have greater artistic abilities (122).

The *Amh* gene is expressed in the mouse hippocampus (CA1: cell body and dendrites CA3: only the cell body) and it is the only known source of cerebrum- derived AMH found in cerebrospinal fluid (21). AMH is present in both female and male individuals’ hippocampus, with discordance 1.7-fold higher in favor of females. AMHR2 levels are comparable between sexes within these brain regions. Exogenous AMH increases long-term synaptic plasticity and synaptic transmission of the hippocampus neurons (21). These two features of the hippocampus neurons are impaired in Alzheimer’s disease and accelerated aging (123, 124). Thus, AMH is considered a factor regulating the synaptic transmission in the hippocampus and with its paracrine/autocrine function it may influence processes, such as learning and memory (21) (**Figure 4**).

## 7 AMH, the ovary and PCOS

AMH inhibits FSH-dependent follicular growth (80), since AMH influences factors increasing the sensitivity of GCs to FSH (9). AMH levels decrease in healthy women as the follicle grows, which increases the sensitivity of GCs to FSH (9) (**Figure 4**). Furthermore, AMH lowers the activity of gonadotropin-dependent aromatase in human GCs by inhibiting *FSHR* (follicle stimulating hormone receptor) gene expression (9). However, based on a more recent study using infantile mice, AMH does not alter *FSHR* expression, but in its presence FSH cannot stimulate aromatase (96). Surprisingly, AMH has no effect on inhibin A and VEGF (vascular endothelial growth factor), which are controlled by LH (9, 125, 126). At high concentrations, AMH does not affect inhibin B in GCs obtained from small and large follicles, although at low concentration it increases inhibin B production in GCs of small follicles (9). Inhibin B is positively regulated by FSH (127, 128), which may explain the observed relation.

LH has no effect on the *AMH* gene expression level in normo-ovulatory women but induces it in luteinized GCs of women with PCOS (129). In healthy women, LH reduces the *AMHR2* gene expression in luteinized GCs but has no effect on women with PCOS (129). Also, a difference in AMH concentrations has been observed during the ovulatory cycle and anovulatory cycle in healthy eumenorrheic women (130). AMH was significantly higher in women undergoing anovulatory cycles at corresponding times of the cycle (130).

**Figure 5:**
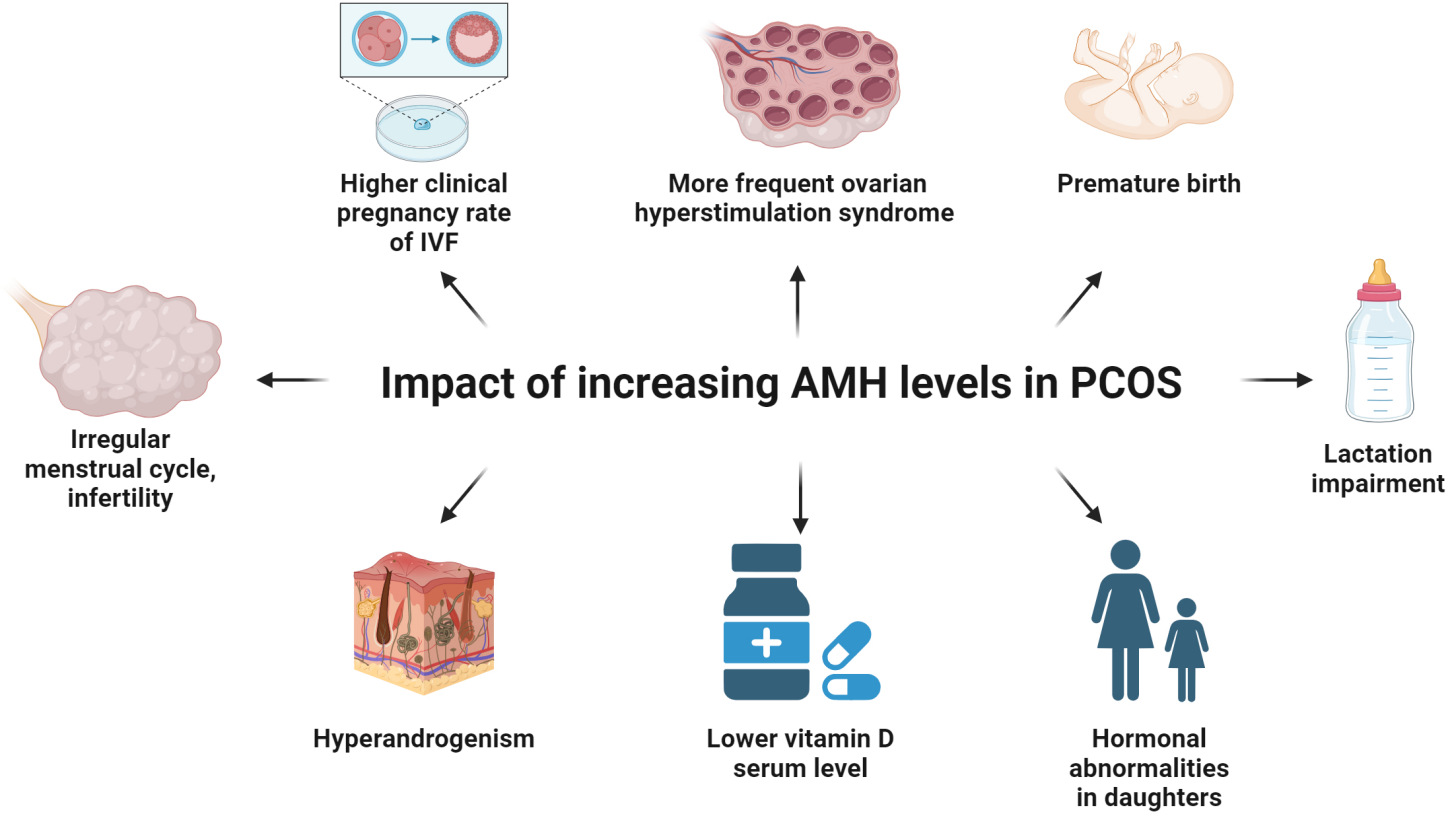
The influence of the increasing levels of AMH on women with PCOS. Increasing levels of AMH in PCOS women inhibit the enzyme aromatase, increase the production of LH by the pituitary gland and decrease the influence of FSH on ovarian tissue, thus leading to irregular menstrual cycle and infertility. IVF procedures among patients with elevated AMH concentration may result in a higher clinical pregnancy rate and a more frequent ovarian hyperstimulation syndrome. Pregnant women with PCOS showing a high level of AMH are at risk of preterm labor in the third trimester as compared to the group of healthy women. Breastfeeding is impaired in the group of women with PCOS because not decreasing AMH concentration impairs the preparation of the breast tissue for the lactation period. Young daughters (age 4-16) of PCOS women show abnormal concentration of FSH, AMH, testosterone and FAI, in comparison to the offspring of healthy women. The level of vitamin D is inversely proportional to the concentration of AMH and positively correlated to the level of testosterone, DHEA-S and FAI in the PCOS women group.

AMH appears to play an important role in the pathogenesis of PCOS (9), which is characterized by chronic anovulation, hyperandrogenism and distinctive ovarian morphology (9, 131) (**Figure 5**). In women with PCOS, the plasma concentration of AMH is 2-3 times higher (132) or even 12 times more than the norm (133) (**Figure 3**). The concentration of AMH increases in line with the number of ovarian follicles (133). Hence, the concentration of AMH inside the ovarian follicle is higher in patients with PCOS (134). Diabetes is a factor reducing AMH levels in women with PCOS, possibly because of vascular damage of OR (135, 136).

In women with PCOS, there is a positive correlation between the AMH concentration and the LH/FSH ratio as well as the level of LH, testosterone, dehydroepiandrosterone sulfate (DHEA-S), total cholesterol, low-density lipoprotein (LDL) and the FAI (free androgens index), which is the quotient of free testosterone and sex hormone binding globulin (SHBG), fasting insulin level and HOMA-IR (homeostasis model of assessment-insulin resistance) (133, 137, 138). A correlation between the AMH concentration and BMI (body mass index), FSH and fasting plasma glucose level was not found (137, 138). Moreover, some studies do not confirm the correlation between AMH and HOMA-IR (139).

AMH levels vary significantly across the four PCOS phenotypes which are distinguished by the presence of features, such as anovulation, hyperandrogenism, and polycystic ovarian morphology (138, 140). When all of the above features are present, it indicates phenotype A (138, 140). The absence of hyperandrogenism in the presence of anovulation and characteristic ovarian morphology means phenotype D (138, 140). The highest value of AMH is observed in patients with all symptoms of PCOS (phenotype A), while the lowest in phenotype D, in which no hyperandrogenism is observed (138).

As AMHR2 is expressed in the adrenal gland (29), there may be a connection between the AMH concentration and androgens in PCOS. However, it is difficult to find a clear connection between these facts. There are groups among PCOS patients, especially those with lower BMI, that exhibit lower concentrations of androgens and still high levels of AMH (141, 142). On the other hand, women with PCOS that show lower levels of androgens more often need the help from specialized infertility clinics than their peers with higher levels of these hormones (141, 142). This may be related to increased engagement of small follicles in development by androgens (133, 143, 144). It was postulated that elevated levels of AMH may play a crucial role in the course of PCOS. Therefore, it is worth considering the use of AMH antagonists in the treatment of infertile women with PCOS (145). The proposed mechanism of increasing AMH levels of in PCOS women is based on the direct excitability of GnRH neurons (100). AMH could cross the blood–brain barrier at the level of the *organum vasculosum* of the *lamina terminalis* and the median eminence (100). An indirect effect could occur at the level of the median eminence through tanycytes and vascular endothelial cells (100). This may result in a specific pattern and frequency of the released GnRH and could lead to LH secretion in much higher concentrations than normally (100).

It is likely that the abnormal design of follicular development has a genetic component (146, 147). Prepubertal daughters of women with PCOS (newborns and 4-7 years of age) show increased AMH and reduced FSH levels compared to their healthy peers (146) (**Figure 5**). Peripubertal daughters (age 8-16) of PCOS mothers have an increased level of AMH, testosterone and FAI, in comparison to the offspring (with the same body mass index, age and breast Tanner stage) of healthy women (147) (**Figure 5**). Also, the average ovarian volume is higher in the group of peripubertal girls who are daughters of PCOS mothers (147).

The regulatory SNP rs 2002555 is located at a binding site of transcription factors MYB (MYB proto-oncogene, transcription factor) and MYC (MYC proto-oncogene, BHLH transcription factor) in the regulatory region of the *AMHR2* gene (148). It was suggested that this SNP may be responsible for the development of PCOS. However, the conducted pooled analysis (3 studies, 5 different loci) did not show any significance in the context of predisposition to the development of PCOS in the case of the SNP within *AMHR2* gene regulatory region (132). Only PCOS women who are the homozygotes at SNP rs 2002555 (GG) have decreased LH and prolactin levels and a lower LH/FSH ratio than other PCOS women (149). There is also no association (in the pooled analysis) between the SNP gene and the development of PCOS (4 studies, 5 different loci) (132). Genome-wide association studies (GWAS) assessing the genetic predisposition to the development of PCOS in the Chinese Han population identified 20 SNPs, 5 of which have been shown to be closely correlated (150, 151), within 11 GWAS loci, but none of them were located in relation to the genes *AMH* or *AMHR2* (150–153). Similarly, among loci predisposing women to the development of PCOS in the European population, a significant correlation was detected for 4 SNPs, but again none of them were located close to the *AMH* or *AMHR2* gene (154, 155).

Pregnant women diagnosed with PCOS, after the ovulation induction with letrozole or clomiphene (but non-IVF procedure) and a high AMH concentration in the first trimester (especially > 9.3 ng/mL), are at risk of preterm labor in the third trimester as compared to the group of women who underwent the induction of the ovulation but PCOS was not recognized (156, 157) (**Figure 5**). A similar relation with childbirth before 37 weeks of gestation is observed for increased AMH levels at the turn of the first and second trimester (158). This may be justified with a limitation in stretching of the uterus caused by the inhibitory effect of AMH on myometrial cells, as well as a direct negative impact of a high AMH concentration on decidualization or placentation (156, 158). During normal pregnancy of healthy women, the concentration of AMH decreases gradually until delivery, while in women with PCOS, the decrease is insignificant, maintaining notably higher values throughout pregnancy (159, 160). As it is known that the preparation of the breast to the lactation period depends on the decreasing level of AMH, it is not surprising that breastfeeding is impaired in the group of women with PCOS (27, 161, 162) (**Figure 5**).

## 8 AMH and menopause

AMH plays a role in regulating the recruitment of primordial follicles (148), *i.e.*, the hormone may influence the time of menopause. Even though SNPs of the *AMH* gene (Ile49Ser; rs10407022) and *AMHR2* gene (482 A→G; rs2002555) are associated with a higher concentration of estradiol in the follicular phase of healthy normo-ovulatory women (163), it is difficult to find a strong correlation between the time of menopause and genetic variations of the *AMH* gene. However, the SNP of the *AMHR2* gene with the SNP rs20025555 (G/G) means an earlier (on average by 2.6 years) menopause than with the A/A genotype (148). Nonetheless, this is true only in the normo-ovulatory nulliparous group of women (148).

On the other hand, a simulation model for female hormonal regulation based on 16 non-linear differential equations with 66 parameters indicates that exogenous AMH may delay menopause (164). Thus, mathematically confirmed the administration of exogenous AMH in a dose of 5 ng/mL between the age 25 and 35 delays the time of menopause by 2 years (164). An increased dose of AMH (20 ng/mL) postpones the menopause even by 5 years (164). This is another proof of stabilization of the pool of primordial follicles by AMH. A different study suggests it is possible to predict the onset of menopause based on a cut-off point of AMH levels (below 0.1 ng/mL) using mathematical modeling of the AMH level decreasing with age (165) (**Figure 3**). The topic of calculating the time to menopause seems to be more controversial. There are studies indicating the uselessness of AMH decline rate for predicting early menopause (166). However, in the literature on the subject, the usefulness of AMH concentration determinations is more often indicated. AMH rate of change estimated together with AMH baseline level in healthy premenopausal women is particularly important in predicting early menopause in the age group of 35-39 years (167). In the context of predicting not only early menopause but menopause in general, the advantage of multiple AMH concentration determinations over a single measurement is emphasized (168).

## 9 AMH, artificial reproductive technologies and cryopreservation of ovarian tissue

In women of the reproductive age, AMH is a measure of OR, and its concentration is the most reliable indicator of the success of artificial reproductive technology (ART) (169–171) (**Figure 3**). IVF procedures among patients with elevated (5-10 ng/mL) and ultrahigh (> 10 ng/mL) AMH levels show a higher number of good quality embryos, a higher clinical pregnancy rate and a more frequent ovarian hyperstimulation syndrome (OHSS) (133) (**Figure 5**). Perhaps this phenomenon is related to a significantly higher serum concentration of androgens which usually is observed in the group of women with higher AMH levels (133, 143, 144). Androgens are responsible for the potential and maturation of preantral and small antral follicles (while they are still acquiring the ability to respond to FSH), which are the main source of AMH in women (133, 143, 144). Therefore, it seems that androgens, among others, are responsible for an increase in AMH levels (133). In an animal model of primates, androgen administration increases *FSHR* gene expression in GCs (143). Hence, it can be concluded that AMH is a determinant of greater sensitivity of ovarian follicles to gonadotropins. It is not surprising that AMH as a pretreatment agent before triggering superovulation increases antral follicle count (AFC), (as it was demonstrated in a mouse model) and protects against follicle atresia (172).

Although it would seem that the AMH level is a more objective measurement than the AFC, the superiority of AMH determinations over AFC in the desired ovarian response in IVF protocols could not been confirmed (173, 174). Also, the concentration of AMH in the blood is unlikely to be related to embryo quality (EQ), but a higher concentration of AMH in the follicular fluid seems to be a favorable prognostic factor for top-quality blastocyst and live birth ratio (175, 176). A prospective observational multicenter study revealed that the cut-off value of AMH predisposing to achieving the criteria for hCG (human chorionic gonadotropin) triggering was 4 pM (0.56 ng/mL) but with the live birth ratio only 5.7% per each started cycle (177) The lowest serum concentration of AMH which positively correlated with the live birth was 1.3 pM (0.18 ng/mL) (177). However, this is not the basis for not attempting to use ART at concentrations of AMH below those thresholds (177). Hence, AMH alone should not be the only factor considered when deciding to initiate ART procedures (178). It is important to individualize each case and correlate AMH with AFC and biometric features (178) (**Figure 3**). However, the usefulness of AMH concentration assessment together with body weight in the prevention of OHSS in ART protocols is confirmed when daily doses of follitropin delta (a recombinant FSH) depend on the factors mentioned above (179) (**Figure 3**).

**Figure 6:**
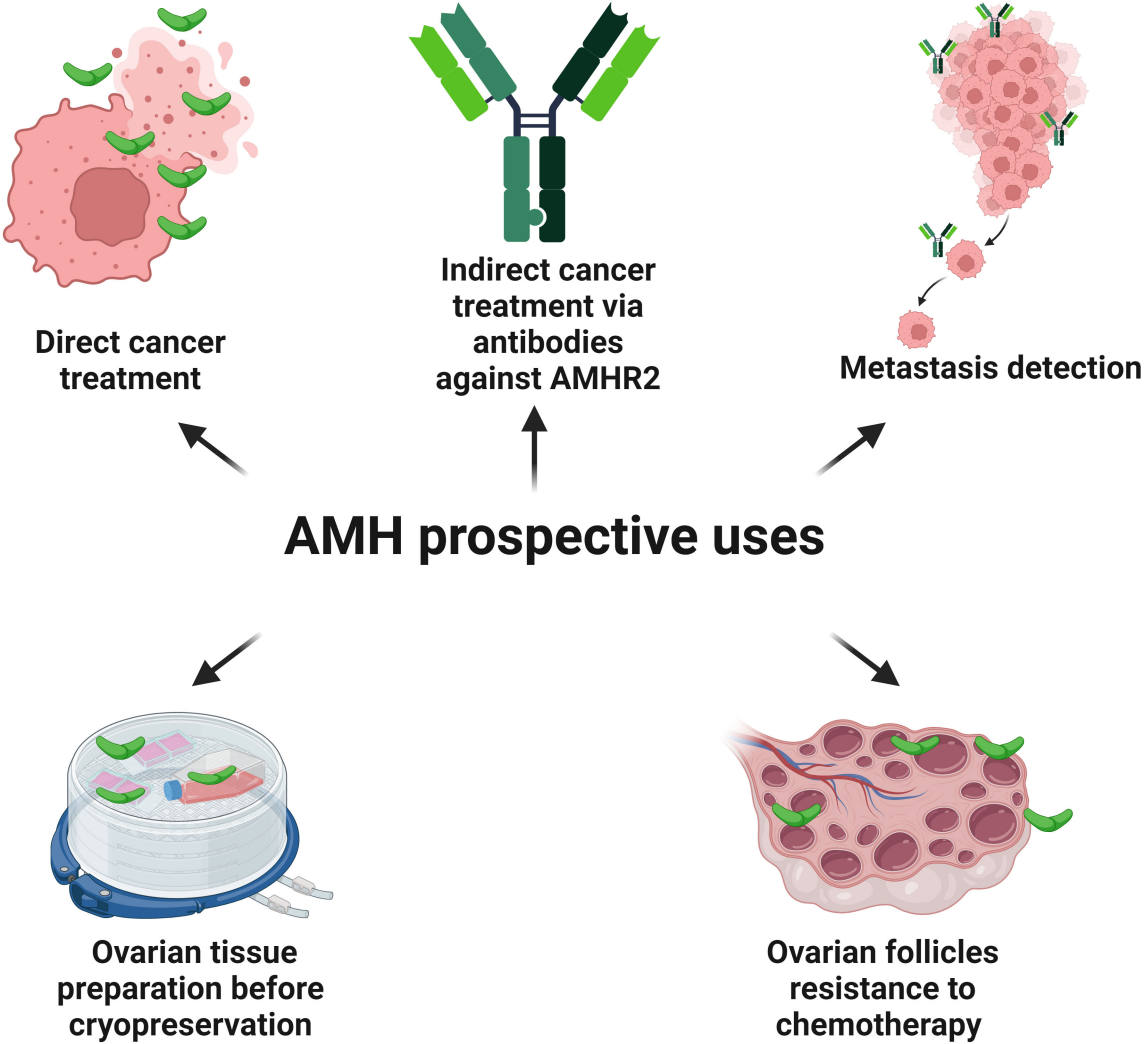
Possible future utilities of AMH and antibodies against AMHR2. AMH has the potential to inhibit the cell cycle and induce apoptosis in the cells of the different types of cancers possessing AMHR2. Antibodies conjugated with a radioactive isotope and targeting AMHR2 destroy cancer cells in an animal model. Also, a monoclonal antibody against AMHR2 (murlentamab), switches the pro-cancer nature of TAMs towards the anti-cancer action by activating specific immunological mechanisms destroying tumor cells (phase I and II clinical trials). Conjugated anti-AMHR2 antibodies with the radioactive isotope zirconium are useful in the detection of intraperitoneal EC metastases (animal model). Also, in an animal model, AMH shows a protective and positive effect on the ovarian reserve, before the use of chemotherapy, which is toxic to the ovarian tissue. As AMH stabilizes the pool of the primordial follicles, it could increase the resistance of ovarian tissue to the harmful condition of cryopreservation procedures.

Also, in terms of the decision to qualify the patient for the procedure of ovarian tissue cryopreservation before cancer therapy, the serum AMH level is one of the main factors taken into consideration (180) (**Figure 3**). It is worth remembering that the clear relationship between AMH level and OR applies only to patients over 25 years of age (181). Methods of ovarian tissue cryopreservation are expected to improve in the future, in order to increase the viability of the retransplanted ovarian tissue, maintaining the appropriate concentration of sex hormones affecting the quality of life as well as increasing the chances of achieving pregnancy and live birth (182, 183). Based on the role of AMH in stabilizing the pool of primordial follicles, AMH could become a useful substance in cryopreservation procedures of the ovarian tissue in the future (**Figure 6**). In an animal model, AMH shows a protective and positive effect on OR, before the use of chemotherapy, such as carboplatin, doxorubicin or cyclophosphamide, which is toxic to the ovarian tissue (180, 184). In *ex vivo* studies, ovarian tissue exposed to the toxic metabolite of cyclophosphamide shows greater resistance to the chemotherapeutic agent when AMH is added (185) (**Figure 6**).

## 10 AMH, AMHR2, cell cycle and cancer

The presence of AMHR2 in the tissue is a crucial aspect of the modern approach to targeted oncological therapy using the anti-cancer properties of AMH. A promising direction for the use of AMHR2 in diagnostics and therapy is the application of conjugated anti-AMHR2 antibodies with the radioactive isotope zirconium (^89^Zr) in the detection of intraperitoneal EC metastases and in radioimmunotherapy of EC with the radioactive isotopes lutetium (^177^Lu) and bismuth (^213^Bi) (136) (**Figure 6**).

In the tumor microenvironment, a low fucosylated antibody against AMHR2, murlentamab (GM102), switches the pro-cancer nature of tumor-associated macrophages (TAMs) towards the anti-cancer action by activating immunological mechanisms leading to the destruction of tumor cells (186–189) (**Figure 6**). Initially, TAMs contribute to the tumor progression by producing anti-inflammatory chemokines. However, reprogrammed by GM102, they acquire the features of M1-type anti-cancer macrophages by stimulating cytotoxic T cells (CD8^+^), antibody-dependent cell-mediated cytotoxicity (ADCC) and antibody-dependent cellular phagocytosis (ADCP) (189–191). The promising effect of GM102 on AMHR2-expressing tumors was already reported in a preclinical study in cynomolgus monkeys using a xenograft of human ovarian cancer (192). GM102 used in phase I clinical trial in a group of women with ovarian cancer had an impact on the proportion of subsets of monocytes in peripheral blood (193). Then, in the first-in-class trial among 27 women with gynecological cancers expressing AMHR2, it was shown that GM102 is well tolerated at all doses and decreases the tumor growth rate in 47% of patients (194). This effect was achieved through the activation of monocytes, neutrophils and lymphocytes (194). The next first-in-class trial of GM102 with cisplatin and paclitaxel was conducted on the group of patients with ovarian, cervical and endometrial cancers. However, better response was noted for treatment combined with cisplatin and paclitaxel when compared with GM102 alone (195). No dose-related toxicity and only weak side effects related to applications of GM102 were observed. The activation of classical monocytes, T cells and neutrophils in blood was detected together with changes in TAMs (195). Phase II trial of GM102 alone or in combination with trifluridine/tipiracil (FTD/TPI) was conducted on patients with colorectal adenocarcinomas (196). Better response to treatment increased and was associated with a higher number of cancer cells with AMHR2 expression (196). Paired biopsies revealed the activation of tumor immune microenvironment (CD16 macrophage) and phagocytosis. GM102 together with FTD/TPI activated antigen-presenting cells (CD86) and CD8^+^ T cells (196). In peripheral blood, GM102 stimulated monocytes (CD69^+^) and neutrophils (CD64^+^) as a single factor or with FTD/TPI (126). Taking into account phase I and phase II clinical trials of patients with colorectal and ovarian cancer treated with GM102, it was shown that in the case of colorectal cancer the number of blood monocytes CD69^+^ increases, while the number of CD69^+^ activated-regulatory T cells decreases (189). Also, the blood concentration of two substances presented as critical immune modulators and survival predictors, such as the interferon-inducible chemokines CXCL (C-X-C motif chemokine ligand) 9 and 10, increases (189, 197). Paired biopsies revealed that GM102 changes the proportion of macrophages in favor of cells engaged in the ADCC/ADCP process with activation of natural killer (NK) cells and CD8^+^ cells (189). In the case of ovarian cancer, the number of CD8^+^ T cells expressing inducible co-stimulator molecule increases (189). An experiment conducted *in vitro* with the culture of human ovarian cells with the presence of AMHR2 and microenvironment with TAMs showed that GM102 polarizes the CD4^+^ T cells towards T_H_ (T helper) 1 cells and CD8^+^ T cells profile (189). Interestingly, *in vitro* GM102 positively cooperates with pembrolizumab, an anti-PDCD1 (programmed cell death 1) antibody, which is useful in cancers with microsatellite instability (Lynch syndrome) with profiling lymphocytes towards T_H_1 cells (189, 198). In the animal model, GM102 together with pembrolizumab promote changes observed in the ADCC/ADCP process. Thus, it may be advisable to combine the treatment of specific cancers (expressing AMHR2 and microsatellite instability) with these two agents (189, 198) (**Figure 6**).

Apart from the development of tissues and organs as well as wound healing, cancers are the major cause of EMT (39, 199). Theoretically, during EMT AMHR2 could be present on non-gynecological solid tumor cells. It was confirmed that AMHR2 is expressed in hepato-carcinomas (HCC), colorectal (CRC), non-small-cell lung (NSCLC) and renal cancer cells (RCC) (29). *AMHR2* expression was also detected in melanoma and head and neck cancer cells. Interestingly, in NSCLC AMHR2 expression was more common in women (67%) than in men (30%) (29). The studies conducted in animal models comparing the efficacy of GM102 and standard treatment of HCC and CRC, sorafenib and irinotecan, respectively, revealed that GM102 has similar efficacy to sorafenib and irinotecan, but treatment with GM102 shows better tolerability (29) (**Figure 6**).

Not only the targeting of specific antibodies against AMHR2 in tumor tissue should be considered in future oncotherapy, as the anti-proliferative activity of AMH manifests in two aspects the influence on the cell cycle and the regulation of apoptosis. Thus, AMH plays a regulatory role in endometriosis cells and other gynecological neoplasms (25–27, 30–32). AMHR2 is expressed in nearly 70% of human ovarian endometriomas cells (30). Other benign ovarian tumors express AMHR2 in approximately 45% of all cells (200). Following the addition of AMH to the culture of endometrial cells, the survival of those cells is significantly reduced to 68%, compared to the control samples (30). An increase in the percentage of cellular DNA from the G_0_G_1_ and sub-G_0_G_1_ phases indicates that AMH has an inhibitory effect on the cell cycle by suppressing cells in the G_1_ phase of the cell cycle (30). Interestingly, AMH increases the level of the cyclin-dependent kinase (CDK) inhibitor p21, the Rb family factors p107 and p130 as well as the transcription factor E2F2 (30). On the other hand, the level of E2F1 decreases after AMH administration (30). Short peptides of cyclin binding domains of the proteins p21, p107 and p130 compete in binding to CDK2, causing its inhibition (30, 201, 202). E2F1 and E2F2 are transcription factors with a dual nature, since they promote the cell cycle progression but also regulate apoptosis and DNA repair (203–205). An increased level of the apoptosis-inducing factor (AIF), the active form of caspase 9, cleaved PARP (poly ADP-ribose polymerase) and a decreased level of caspase 3 after AMH addition to endometriosis cells may prove the apoptotic activity of AMH (30). However, it seems that the mechanism of the pro-apoptotic action of AMH is different in endometriosis and gynecological malignancies.

**Figure 7:**
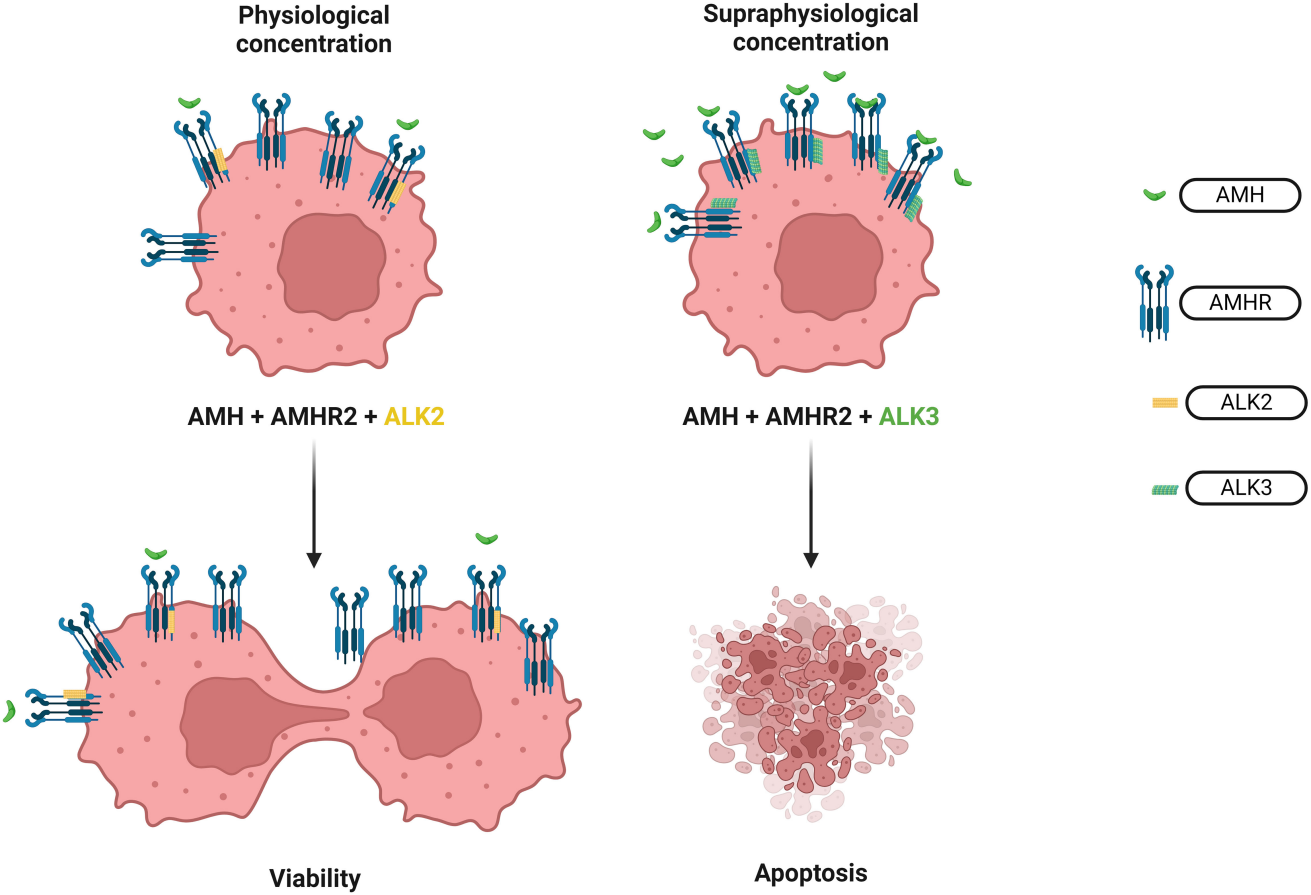
Effect of different AMH concentrations on ovarian cancer cells. The opposite effect of physiological and supraphysiological AMH concentration on the survival of the ovarian cancer cells. In physiological concentration, AMH works through ALK2 recruitment thus increasing the survival rate of the cell colonies of the high serous ovarian adenocarcinoma. Meanwhile, supraphysiological concentration of AMH activates ALK3 resulting in apoptosis of ovarian cancer cells.

In the ovarian cancer cell line OVCAR-8, AMH acts mainly through a mechanism that depends on the CDK inhibitor p16 (31). Increased expression of p16, p21 and E2F1 proteins as well as decreased levels of p130 have been demonstrated (31). One of the most convincing evidence of the anti-tumor activity of AMH is its additive effect on ovarian serous cancer with paclitaxel and cisplatin and its synergistic effect with rapamycin and doxorubicin (28) (**Figure 6**). Also, exogenous AMH concentrations beyond the physiological values reduce the cell survival of the high-grade serous adenocarcinoma of the ovary (206) (**Figure 7**) Taken together, recombinant human AMH inhibits cell colony growth in most advanced ovarian cancer cell lines (207) (**Figure 7**).

Physiological concentrations of endogenous AMH play a surprising role in the context of oncology as they increase the survival rate of the cell colonies of the high serous adenocarcinoma of the ovary tumor, sex cord-stromal tumor and the granulosa cell tumor (206) (**Figures 6**, **7**). This mechanism involves inducing phosphorylation of the PI3K/AKT/mTOR pathway through ALK2 recruitment (207) (**Figure 7**). Even partial AMH depletion or inhibition by specific antibodies reduces the viability of cells of ovarian cancers, decreasing phosphorylation of the PI3K/AKT/mTOR cascade and increasing PARP and caspase 3 cleavage (207). However, a supraphysiological concentration of exogenous AMH engages the ALK3 pathway and decreases the survival of ovarian cancer cells (208) (**Figure 7**). Similarly, to the phenomenon described above, the physiological and supraphysiological AMH concentrations affect the survival of Sertoli cells (209). The use of bispecific antibodies against ALK2 and AMHR2 appears to be more potent in the context of anti-cancer activity than bispecific antibodies ALK3 and AMHR2 (208) (**Figure 6**).

**Figure 8:**
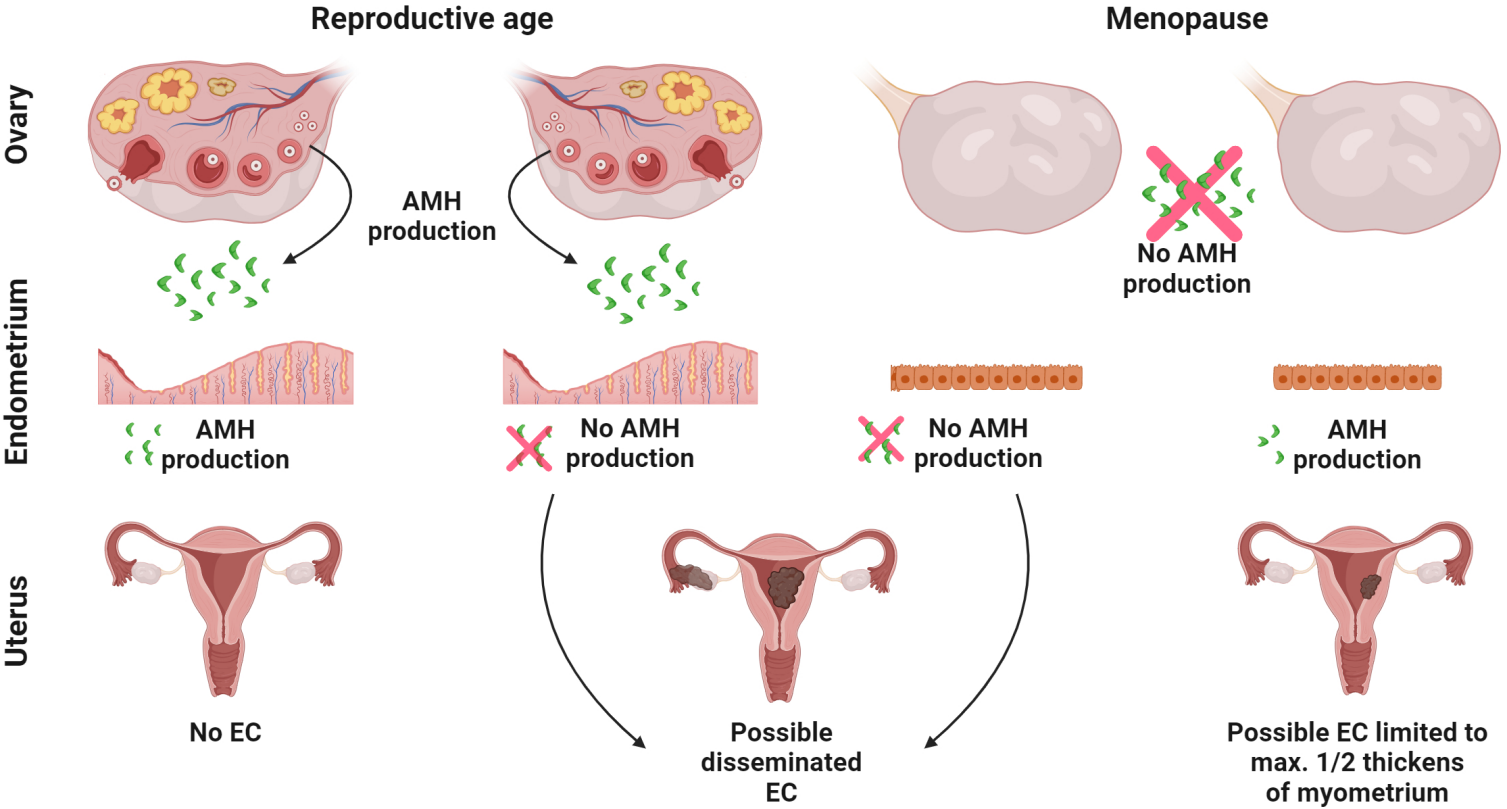
Crosstalk between AMH and endometrial cancer. The putative interaction between local endometrial expression of AMH and ovarian source of AMH on the development of EC. AMH from two sources acting simultaneously: the endometrium (auto/paracrine activity) and the ovaries (endocrine activity) protects against EC. When endometrial origin AMH is not produced even in the presence of ovarian origin AMH, the disease may develop. When there is a lack of ovarian source AMH, but the endometrium still produces AMH, the EC is limited to a maximal ½ thickness of the myometrium. All figures were created with BioRender.com.

Apart from ovarian cancer, EC, the most common neoplasm of the female reproductive organs, also appears to be a suitable target for the use of AMH (25, 45). In the cell line AN3CA of EC, the inhibitory effect of AMH on proliferation is confirmed by increased levels of p130 and p107 (25) (**Figure 8**). Incubation of EC cells with AMH results in a reduced levels of the transcription factor E2F1, yet it does not affect the level of E2F2, E2F3 or E2F4 (25). Moreover, AMHR2 is present in all types of histopathological EC (33). AMHR2 is also found in all EC stages according to FIGO classification (33). The expression of AMHR2 in EC is not influenced by BMI, the patient’s age, their parity, the mass of newborns, breastfeeding time, number of miscarriages, number of menstrual years, hormonal status, use of hormonal replacement therapy or the presence of arterial hypertension. The only factor reducing *AMHR2* gene expression in EC is type 2 diabetes (33). Interestingly, women with type 1 diabetes also have decreased AMH levels compared to non-diabetic women (210).

Another issue is the unclear role of intracellular AMH expression in EC. Among the different types of EC, the presence of AMH in EC tissue was confirmed in approximately 10% of cases, diagnosed only after the menopause and never before this moment in female life (22). This applies to EC with a good prognosis well (G1) and moderately (G2) differentiated endometroid adenocarcinoma and clear cell cancer that is characterized by poor prognosis (22). In the latter type of EC, in every case with AMH presence, the neoplastic process is always limited to a maximal ½ thickness of the myometrium (22) (**Figure 8**) A long mean period of lactation (more than 10 months) and a period of sex-hormone activity (time from the first to the last menstruation) longer than 40 years, have a positive effect on the expression of AMH in EC (22). Neither age nor BMI, parity, the mass of the newborn, total breastfeeding time, tumor stage or comorbidities like chronic hypertension or type 2 diabetes affects the expression of AMH in EC (22). Perhaps the intracellular presence of AMH limits the expansion of EC with a poor prognosis (**Figure 8**).

In the C33A cell line of cervical cancer, the pro-apoptotic effect of AMH is manifested by an increase in the levels of p16, p130, p107 and E2F1 proteins (32) (**Figure 6**). AMH also inhibits the growth of the cell line A431 of human vulvar epidermoid carcinoma (211) (**Figure 6**). AMH reducing effect on lung metastases of the human eyeball malignant melanoma cell line OM431 was demonstrated in the mouse model (**Figure 6**). On the other hand, AMH does not inhibit the growth of cell lines of human bladder transitional cell papilloma (RT-4) and human hepatocellular carcinoma (Hep 3B) (211).

AMHR2 expression has been also confirmed in cells originating from healthy breast and prostate tissue, breast fibroadenomas and various breast and prostate cancers as well as their cell lines (26, 27, 212). During pregnancy, epithelial cells of rat mammary ducts proliferate intensively and undergo apoptosis once lactation has finished (26). This process depends, among other factors, on AMH (**Figure 4**). The *Amhr2* gene expression in the rat mammary gland decreases during pregnancy, is at its lowest during the lactation period and increases once again shortly after weaning (26).

The growth of the proximal part of the murine prostate gland occurs in the early stages of development and is dependent on androgens to a limited extent (213, 214). Expansion of the distal part of the prostate takes place after the 15^th^ day of embryonic development, when the AMH level declines (213, 214). This appears to be an androgen-dependent process. AMH inhibits testosterone synthesis *in vitro* and *in vivo* by reducing the expression of the *CYP17A1* (cytochrome P450 family 17 subfamily A member 1) gene, which encodes for a 17α-hydroxylase that converts progesterone to androstenedione (34, 58, 63, 215).

In AMHR2 signal transduction in BC and PC, three distinct subtypes of type I receptors for AMH are involved ALK2, ALK3 and ALK6 (26, 27) AMH inhibits the growth of BC cells that express ESRs (*e.g.*, T47D) and those that do not (*e.g.*, MDA-MB-231) (26) (**Figure 6**). Flow cytometry revealed an increase in the number of cells in the G_1_ phase by 10-16%, compared to cells that were not incubated with AMH or treated with biologically inactive AMH (26, 27). In addition to disrupting the cell cycle, AMH induces apoptosis in T47D cells (26) (**Figure 8**). There was a 3-fold increase in the concentration of caspase 3 and a 3-fold increase in the early apoptosis marker annexin V, compared to the cells there were not treated with AMH (26, 27). In AMH-treated BC (T47D) and PC- LNCaP (cells of androgen-sensitive human prostate adenocarcinoma) cells, the NFκB signal transduction pathway is activated (27, 216). In addition to AMH, this pathway is also activated also by free radicals, UV radiation, antigens and pro-inflammatory cytokines like TNF (tumor necrosis factor) and IL1β (interleukin 1β) (217). In both BC and PC, p50/p65 heterodimers are induced. However, p65/p65 homodimers are activated only in the BC line, and p50/p50 are present only in the PC cell line. In contrast, the biologically inactive, non-cleaved form of AMH does not activate the NFκB pathway (26, 40). T47D and LNCaP cell lines treated with AMH show induction of the *RGS1* (regulator of G protein signaling 1) gene expression (26, 27). As the promoter of the *RGS1* gene has binding sites for both NFκB and p53, it plays a regulatory role in the context of the cell cycle, differentiation and stress response (218). Additionally, selective expression of *RGS1* splice variants were found in T47D cells after incubation with AMH (26). There was no expression of variant *RGSL* (responsible for the survival of the cell) and anti-apoptotic NFκB-induced factors, such as A20 and c-IAP2 (26). Cells of the ESR-negative BC cell line MDA-MB-231 react similarly to incubation with AMH. The NFκB pathway is activated, with the presence of the p50 and p65 subunits and the *RGS1* gene. Transcripts of both splice variants: *RGS1S* and *RGS1L* have been reported, but biologically relevant levels are reached only by *RGS1S*, resulting in cell cycle inhibition in approximately 50% (26).

Interestingly, only proper regulation of NFκB with an optimal degree of inflammation or apoptosis, including apoptosis induced by anti-cancer drugs, leads to the desired effect from the point of view of the organism’s interests (219, 220). Dysregulation of NFκB signaling leads to cancer, metastasis, chronic inflammation or autoimmune diseases (219). Upregulation of NFκB, which happens in various cancers, is related to the presence of cytokines in the tumor microenvironment that activate the NFκB pathway leading to an increase in anti-apoptotic molecules (221, 222). Understanding the effects of NFκB in different circumstances makes it possible to properly interpret the results of activation and inhibition of the NFκB pathway in different types of BC. Mammogenesis and lactogenesis are regulated, among others, by sex hormones and modulators of their signals (223). One of them is RANKL (receptor activator of nuclear factor kappa beta ligand) (223–225). Excessive exposure of progesterone receptor (PGR) positive cells to gestagens causes overproduction of RANKL, which subsequently activates RANKL receptor in PGR negative cells (223, 226). Ultimately, this leads to upregulation of NFκB, downregulation of the CDK inhibitor p21 and consequently to cancerous transformation of the breast tissue (227–230). The usefulness of the human monoclonal antibody denosumab in breast cancer, which blocks RANKL, has already been evaluated (231). The above-mentioned pro-apoptotic response of BC cells to AMH, activating NFκB and then the synthesis of *RGS1*S, would be limited by excess estrogens, which reduce the density of AMHR2 preventing the beneficial activity of AMH (232). Taken together, there is an intensive crosstalk between sex hormones and BC.

Despite anti-cancer activity of AMH and the fact that the anti-proliferative effect of AMH on the mammary gland is gradually reduced during physiological pregnancy to ensure proper lactation, there is no clear evidence that increased or decreased plasma AMH concentrations have a significant relationship with BC (161, 162, 216, 232). The literature on the subject is inconsistent and links lower AMH concentration with a higher risk of BC as well as a positive correlation of AMH concentration with BC, and on the other hand, the lack of the relationship or the correspondence of BC only with the lowest and highest quartiles of AMH level (233–236). The relationship of AMH with BC caused by *BRCA1*/*BRCA2* (BRCA1/2 DNA repair associated) mutation is also described differently in various studies (237, 238). In conclusion, it appears that plasma AMH concentrations in patients, even with PCOS, are significantly lower than those used in the studies describing the effect of AMH on the BC tissue in *in vitro* conditions (232). Thus, it is difficult to reach a final statement on the influence of AMH serum levels on BC (232). It is worth mentioning the significant potential utility of AMH level assessment in the context of ovarian function loss after chemotherapy for BC. Women with BC who are over 40 years of age and who have undergone anthracycline- and taxane-based chemotherapy and have undetectable AMH levels after 6 months of treatment have very likely irreversibly lost ovarian function (239). Analysis of AMH levels in this group of patients would allow avoiding therapy with a GnRH agonist aimed at inhibiting estradiol production, which is associated with serious side effects (239).

There is also another connection between RANKL, NFκB and AMH. AMH inhibits RANKL-dependent osteoclast differentiation by preventing the degradation of the IκB (inhibitor of nuclear factor kappa B) protein (240). Under the influence of AMH, the expression of osteoclast differentiation markers (*FOS*, *NFATC1*, *ACP5*) is reduced (240) (**Figure 4**). However, it does not affect osteoblast differentiation dependent on BMP2, which is another member of the TGFβ family (240). There is no expression of AMH in osteoclasts and osteoblasts (240). Taking into account the inhibition of osteoclast differentiation, the usefulness of AMH in premenopausal and perimenopausal age as a marker of low bone mineral density is considered (241–243). Therefore, there may be a functional crosstalk between vitamin D and AMH. The serum level of 25-hydroxyvitamin D_3_ is inversely proportional to the concentration of AMH and positively correlated with SHBG. However, this applies mainly to women with PCOS (244) (**Figure 5**). On the other hand, vitamin D_3_ supplementation in normo-ovulatory women and women with reduced OR increases the AMH level, having a positive effect on the *AMH* gene expression, without affecting the number of antral follicles (245, 246).

## 11 Concluding remarks

In summary, the mechanism of action of AMH as well as involved the signal transduction pathways are tissue-specific. Despite numerous studies and the knowledge gained, further investigations concerning this glycoprotein are needed, since the full potential of AMH is still obscure. However, the modulating effect of AMH on the recruitment of ovarian follicles and the effect of its concentration on the result of IVF is of great interest to fertility specialists. Also, being aware of its important role in the process of the cell cycle inhibition and inducing apoptosis, AMH and its receptor AMHR2 raise great hopes for future applications in oncology.
